# Canine Mammary Mixed Tumours: A Review

**DOI:** 10.1155/2012/274608

**Published:** 2012-10-21

**Authors:** Geovanni Dantas Cassali, Angélica Cavalheiro Bertagnolli, Enio Ferreira, Karine Araújo Damasceno, Conrado de Oliveira Gamba, Cecília Bonolo de Campos

**Affiliations:** ^1^Laboratório de Patologia Comparada, Departamento de Patologia Geral, Instituto de Ciência Biológicas, Universidade Federal de Minas Gerais, Avenida Antônio Carlos 6627, 31270-901 Belo Horizonte, MG, Brazil; ^2^Fepagro Saúde Animal, Instituto de Pesquisas Veterinárias Desidério Finamor (IPVDF), 92990-000 Eldorado do Sul, RS, Brazil

## Abstract

Mammary mixed tumours are the most frequent neoplasias in female dogs. In humans, mixed tumours are frequently found in the salivary glands and are known as pleomorphic adenomas. In addition to their histomorphologic similarities, mixed tumours and pleomorphic adenomas have the potential to become malignant and give rise to carcinomas in mixed tumours and carcinomas ex-pleomorphic adenoma, respectively. The factors associated with malignant transformation are still poorly known in the case of canine mixed tumours. However, this form of neoplasia tends to be associated with a better prognosis than other malignant histological types. This paper discusses the main features associated with female canine mammary mixed tumours.

## 1. Introduction

Mammary tumours are the most frequent neoplasia in female dogs; therefore, these tumours represent a serious problem in veterinary medicine [1]. Mixed tumours are one of the most common tumour types in the female canine mammary glands. These tumours exhibit a complex histological pattern because they comprise elements from the epithelium and the mesenchyme and have the capacity to undergo malignant transformation, thereby giving rise mainly to carcinomas and less frequently carcinosarcomas and sarcomas in mixed tumours [2, 3].

Defining the origin of the several cellular elements involved in mixed tumours, as well as the factors contributing to malignant transformation is important in understanding the behaviour and evolution of this type of neoplasia. However, these components of mixed tumours still remain to be elucidate.

This paper discusses the main features associated with the clinical-epidemiological characteristics, histogenesis, malignant transformation, and comparative aspects of female canine mammary mixed tumours.

## 2. Definition/Morphology

**Figure 1:**
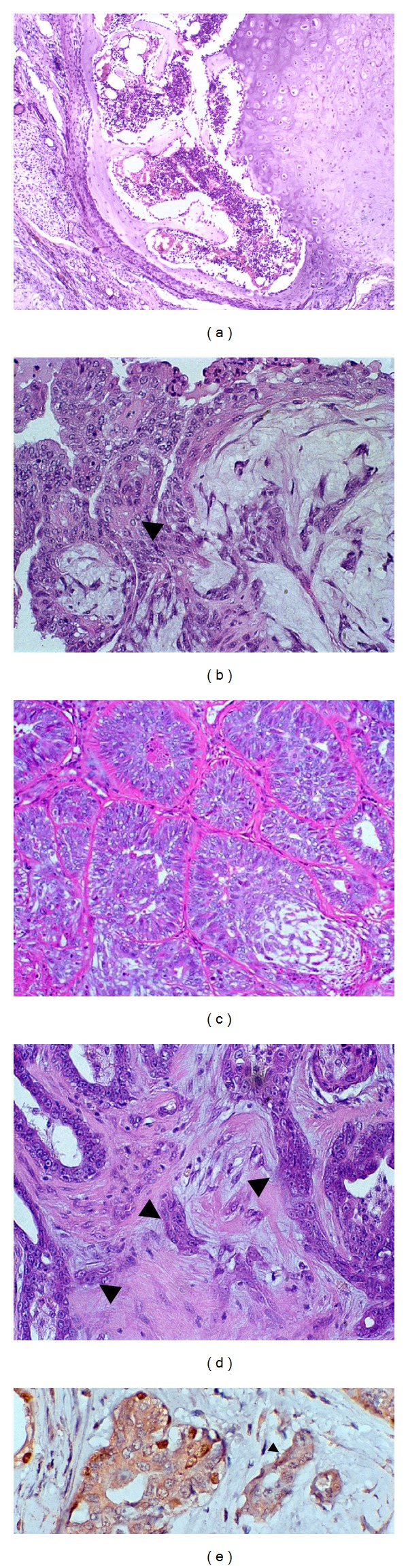
(a) Benign mixed tumor in canine mammary gland presenting chondroid and myeloid metaplasia. HE, 10x. (b) Ductal *in situ* carcinoma in benign mixed tumor in canine mammary gland presenting myoepithelial cells producing myxoid matrix. HE, 40x. (c) Carcinoma in benign mixed tumor in canine mammary gland presenting *in situ* carcinomatous areas and myoepithelial cell proliferation producing myxoid matrix. HE, 20x. (d) Carcinoma in benign mixed tumor in canine mammary gland presenting invasive areas in the adjacent stroma (arrow). HE, 40x. (e) Carcinoma in benign mixed tumor in canine mammary gland presenting absence of myoepithelial cells confirmed through negative p63 expression (arrow) in stromal invasive areas. Immunohistochemical stain with Mayer's haematoxylin counterstain, 60x.

Benign mixed tumours are characterised by the presence of benign epithelial elements (ductal and/or acinar and myoepithelial cells) and mesenchymal cells with cartilage and/or bone formation eventually combined with myxoid fibrous tissue [2] (Figure 1(a)).

The proliferating myoepithelial cells may exhibit a fusiform or stellate appearance, and these cells are often enveloped within an abundant extracellular matrix (myxoid matrix). The cartilage tissue is characterised by nodules or plaques of different sizes, including low or moderate numbers of chondrocytes and chondroblasts rarely exhibiting cellular morphological alterations. When bone tissue is involved, it comprises osteoid matrix-forming osteoclasts and mineralised bone. Certain cases also exhibit bone marrow, including haematopoietic and adipose tissue [4, 5].

A certain degree of pleomorphism and atypia is generally found in these tumours; therefore, the differential diagnosis is often difficult, especially regarding carcinomas in benign mixed tumours. The use of special staining techniques in order to analyse the integrity of the basement membrane allows for a better decision on the benign or malignant nature of this type of tumour [6–10]. 

According to the developing system of classification of carcinomas in mixed tumours, these carcinomas are characterised by a focal or nodular development of malignancy within a primarily benign mixed tumour [2]. 

Initially, the term “malignant mixed tumour” was applied to carcinomas arising in the context of benign mixed tumours. However, several authors used this same term for mixed tumours in which one or both (epithelial or mesenchymal) components were malignant [11, 12]. The term carcinosarcoma was used as synonym of malignant mixed tumour, even in cases without malignant transformation of one of the two cellular components [11].

In the classification scheme proposed by Misdorp et al. (1999), the expression “malignant mixed tumour” was excluded and replaced by “carcinoma in mixed tumour,” which is histologically different from carcinosarcoma. Carcinosarcoma refers to a neoplasia that exhibits the concomitant malignancy of both the epithelial and mesenchymal components and a more aggressive behaviour than the former [2, 13].

In carcinomas in benign mixed tumours, the carcinomatous proliferation might exhibit *in situ* or infiltrative growth, which is suggested by the loss of the continuity of the myoepithelial and basement layers associated with invasion of the stroma by neoplastic cells. In this case the previously benign lesion might eventually be fully replaced by carcinomatous tissue [3]. Thus, the phenotypic assessment of myoepithelial cells is important in the differential diagnosis between these types of lesions (Figures 1(b), 1(c), and 1(d)).

## 3. Clinical-Epidemiological Characteristics

The data on the frequency of benign mixed tumours are difficult to compare, due to divergences among the various classification systems that have been suggested over time [11, 12]. According to the literature, mixed tumours represent 50% to 66% of canine mammary neoplasias [14]. These tumours usually appear in animals 6 to 10 years old, most frequently in females, although they can also affect males [14–16]. Mixed tumours are thought to occur independently from breed [17]. However, Mulligan (1949) found high incidence in the breeds of Cocker Spaniel, Fox Terrier, and Boston Terrier. They more commonly affect the caudal (inguinal, caudal abdominal, and cranial abdominal) glands and occasionally the cranial (caudal and cranial thoracic) glands [14, 17, 18].

Current surveys of cases assessed based on the latest veterinary classification system showed that 40% to 50% of benign tumours are mixed tumours [1, 19]. Data regarding age are scarce, but several studies have reported that benign mixed tumours affect mostly young animals between 3 and 9 years old [20]. 

Carcinomas in mixed tumours represent 10% to 40% of the total number of diagnosed carcinomas [1, 19, 21]. Recent surveys report carcinomas in mixed tumours as the most frequent histological type, representing 20% to 32% of all mammary malignant tumours [1, 22].

Histological characterisation of mammary tumours might be considered an independent prognostic factor. Carcinomas in mixed tumours are associated with an average survival time that is 2- to 3-fold higher than that of other canine mammary carcinomas. Thus, animals presenting with this histological type exhibit a favourable prognosis when compared to animals presenting other types of carcinomas. As such it might be considered a protective factor against the risk of canine mammary tumours associated death [22, 23]. 

One explanation for the better prognosis associated with mixed tumours is related to the expansive growth pattern of these tumours, exhibiting little lymphatic invasion and a low metastatic index [23, 24]. The size of the carcinomatous area included within a canine mammary mixed tumour might also be a factor that affects prognosis [22].

An early and complete surgical excision followed by a histopathological diagnosis is recommended in the treatment of all canine mammary tumours. Surgical delay might result in larger tumours and make their removal more difficult [25]. Moreover, the epithelial component of mixed tumours might exhibit a malignant transformation, thereby giving rise to a carcinoma. As a result, worsening of the clinical progression and consequently prognosis of this disease may occur. Although surgery is able to successfully treat most cases, the identification of cases requiring alternative therapies is mandatory [26].

The biological behaviour of carcinomas in mixed tumours may vary in accordance to the histological type of the malignant epithelial component of the tumour. Because these tumours are inserted within a benign lesion, these neoplasias are expected to be associated with a better prognosis and the affected animals to exhibit longer survival rates.

## 4. Malignant Transformation

Factors determining the malignant transformation of benign mixed tumours have been the focus of some studies [9, 10]. There are few studies on the malignant progression of canine neoplasias [27]. However, in the 1970s, Moulton et al. hypothesised that if mixed tumours had sufficient time to grow, they would undergo malignant transformation. Later, Genelhu et al. (2007) and Bertagnolli et al. (2009) observed molecular alterations that might contribute to the malignant transformation of benign mixed tumours, such as a loss of p63, ΔNp63, and E-cadherin and *β*-catenin expression [10, 20].

A recent study showed that the overexpression of the epidermal growth factor receptor (EGFR) by malignant epithelial cells might occur early in the carcinogenesis of mixed tumours. Moreover, alterations in the expression of this molecule may play a crucial role in the process of malignant transformation in the epithelial component of this histological type [27].

The key morphological characteristic for the differential diagnosis of carcinomas in canine mixed tumours is the presence of areas of invasion or microinvasion within benign mixed tumours [3]. The *sine qua none* condition required to establish stromal invasion is the rupture of the basement membrane and the myoepithelial cell layer surrounding the carcinoma *in situ* [28]. However, in some cases, the visualisation of this area with standard stains, such as hematoxylin-eosin, is extremely difficult. Thus, the use of special stains, such as periodic acid Schiff (PAS) stain, and of antibodies identifying the proteins expressed in the myoepithelial cells, such as p63 (Figure 1(e)), smooth muscle alpha actin, high-molecular-weight cytokeratins, maspin, and calponin, may aid in the identification of invasion foci in mixed mammary tumour of dogs [7–10, 20, 29].

Myoepithelial cells surround the epithelial structure in premalignant lesions and carcinomas *in situ* and serve as a barrier [30] hindering the progression of *in situ* carcinomas into invasive carcinomas [31]. It is believed that this suppressive ability of the myoepithelial cells depends on their full differentiation and that changes in their molecular expression pattern might result in cell function changes. Undifferentiated myoepithelial cells might promote tumour progression [31]. Bertagnolli et al. (2009) observed *in vivo* that carcinomas evolving within canine mixed tumours exhibited decreased p63 expression, which suggests a loss of myoepithelial cells in this area, thereby favouring the invasive and progressive characteristics of these tumours [10]. However, the mechanisms leading to the interruption of this layer are still poorly known (Man et al. 2003). Studies on human breast neoplasias have shown a reduced expression of the oestrogen receptors and of tumour-suppressive proteins, such as maspin, WT-1, and p63, by the epithelial cells close to areas exhibiting a loss of myoepithelial cells, thereby contributing to the aggressiveness and invasiveness of the tumour [28, 30, 32].

In canine mammary tumours, certain components of the extracellular matrix also seem to participate in the process of malignant transformation. Some authors have reported an accumulation of proteoglycans and chondroitin sulphate in both the stroma around the tumour cells and the matrix produced by proliferating myoepithelial cells [33]. Versican, a type of sulphated proteoglycan, is highly expressed by proliferating fusiform cells and myxoid areas of mixed tumours [34]. Erdélyi et al. (2005) showed that the *in vivo* accumulation of versican in the myxoid matrix is associated with the early differentiation of tissue into cartilage [34]. Moreover, the overexpression of this molecule was observed in the invasive areas of malignant tumours, including carcinomas in mixed tumours, indicating the participation of this proteoglycan in the invasion by tumour cells [35].

## 5. Histogenesis

The origin of the several components of mixed tumours is a subject of long-standing controversy and is not yet fully understood. In the 1940s, Allen (1940) reported 4 cases of canine mammary mixed tumours and, based on their morphological characteristics, this author suggested that the cartilage present in this type of neoplasia is probably derived from adult epithelial cells [36]. Other authors [37, 38] found evidence indicating that cartilage and bone are derived from the stromal connective tissue.

The hypothesis supported by the greatest amount of evidence states that the mesenchymal components originate from myoepithelial cells. The first evidence was based on the analysis of cartilage- and bone-forming cells with the use of histochemical and physical methods, as well as by electron microscopy [39–44].

Immunohistochemical tools enable the definition of the molecular changes in the myoepithelial cells assumed to be involved in cartilage formation and indicate a progressive transition of the myoepithelial cells into mesenchymal cells during cartilage formation. A reduction in the expression of myoepithelium typical markers, such as cytokeratins, p63, smooth muscle alpha actin, and maspin, was observed [6–10] in myoepithelial cells. In addition, the mesenchymal phenotype was confirmed by the presence of vimentin and S-100 [6, 7, 45].

The assessment of the expression of proteins involved in chondrogenesis reinforced the initial evidence. Calponin [29], *β* II tubulin [46], versican and aggrecan [34, 35], collagens [34, 47, 48], 3B3(-) neoepitope [33], bone morphogenetic protein 6 and its receptors (BMP-6) [49–51], and chondromodulin-1 (ChM-1) [51] are expressed in myoepithelial proliferation areas and/or chondrocytes and seem to participate in cartilage formation. Molecules involved in cell-extracellular matrix adhesion, such as tenascin, fibronectin, and the neural cell adhesion molecule (NCAM), apparently contribute to the differentiation of the myoepithelium [52].

A further question thus arises; what might be the relationship between the epithelial and mesenchymal components of mixed tumours? A suggested hypothesis states that these components originate from stem cells with a high capability for divergence. This assumption is grounded on immunohistochemical studies [9, 34] and on the observation that the epithelial and mesenchymal components of mixed tumours are monoclonal [6, 53, 54].

Recently, Ferletta et al. (2011) found cells exhibiting stem-cell characteristics in a line developed from a benign mixed tumour. This finding might represent a step forward in studies of stem cells in canine mammary tumours [55].

## 6. Comparative Aspects

In humans, epithelial tumours associated with production of myxoid or osteochondroid matrix are uncommon in the breast and are associated with an uncertain prognosis. These tumours have been described as metaplastic carcinomas with matrix production. However, this pattern of neoplastic proliferation is frequent in human salivary glands, where the tumours are known as mixed tumours or pleomorphic adenomas [56].

In addition to the histological similarity, mammary mixed tumours in female dogs and human salivary pleomorphic adenomas exhibit other similar features. Both tumour types derive from exocrine glands exhibiting similar architecture, the age of onset when they appear is similar, and malignant epithelial transformation can occur and is mainly associated with rapid growth and recurrence [2, 20, 57–60].

 The occurrence of carcinomas ex-pleomorphic adenomas in the salivary glands is infrequent in humans, but these tumours are usually aggressive and result in distant metastases, similar to observations of human mammary metaplastic carcinomas [59–61].

In carcinomas ex-pleomorphic adenomas of the humans, myoepithelial cells surrounding carcinomatous areas exhibit a reduction in the expression of smooth muscle alpha actin, calponin, cytokeratin 14, CD10, laminin, maspin, and p63 [20, 62]. A similar pattern of antigen expression involving cytokeratins, p63, vimentin, protein S-100, *β*-catenin, and E-cadherin was observed in canine mixed tumours, suggesting that myoepithelial cell proliferation plays an important role in the genesis of these tumours [10, 20, 45]. Another similar pattern of alteration observed in these tumours concerns gene p53 mutations and the accumulation of its protein product [59, 63–65].

Alterations in other proteins involved in the regulation of the cell cycle, such as p21 and c-myc [66], and growth factor receptors, such as HER-2 and EGFR [67–69], and a decrease of adhesion molecules, such as E-cadherin and *β*-catenin, and oestrogen and progesterone receptors [20, 69, 70], have also been observed in both human and canine tumours.

Current comparative studies suggest that the matrix-producing glandular tumours observed in canine and human mammary glands and human salivary glands exhibit the same tumourigenic characteristics. Defining the prognostic and predictive similarities among these tumours might provide better information on the clinical behaviour of these tumours and support the use of a spontaneous canine model in studies of human carcinomas.

## 7. Conclusions and Perspectives

Regarding clinical behaviour, mammary benign mixed tumours occur frequently in dogs and are usually associated with a good prognosis. However, divergences in nomenclature and histological classification over time make it difficult to analyse data on relapses, malignant transformation, and biology of these tumours. Studies focusing on clinical features, malignant transformation, histogenesis, and epithelial-mesenchymal interactions might provide new information required to elucidate the clinical and biological behaviour of this type of tumour in the veterinary medicine setting.

From a comparative perspective, canine mammary mixed tumours and human pleomorphic adenomas of the salivary gland exhibit morphological and molecular similarities, suggesting a similarity in the pathogenic mechanisms involved in malignant transformation and histogenesis.

## References

[1] Cassali GD, Melo BM, Madureira N Mammary gland diagnosis of the laboratory of comparative pathology—UFMG, from 2000 to 2008.

[2] Misdorp W, Else RW, Hellmen E (1999). *Histological Classification of Mammary Tumors of the Dog and the Cat*.

[3] Cassali GD, Lavalle GE, De Nardi AB (2011). Consensus for the diagnosis, prognosis and treatment of canine mammary tumors. *Brazilian Journal of Veterinary Pathology*.

[4] Grandi F, Colodel MM, Monteiro LN, Leão JRV, Rocha NS (2010). Extramedullary hematopoiesis in a case of benign mixed mammary tumor in a female dog: cytological and histopathological assessment. *BMC Veterinary Research*.

[5] Auler PA, Bertagnolli AC, Ferreira E (2011). Myeloid metaplasia in canine mixed mammary tumors: occurrence and characterization. *Veterinary Quarterly*.

[6] Gärtner F, Geraldes M, Cassali G, Rema A, Schmitt F (1999). DNA measurement and immunohistochemical characterization of epithelial and mesenchymal cells in canine mixed mammary tumours: putative evidence for a common histogenesis. *The Veterinary Journal*.

[7] Gama A, Alves A, Gartner F, Schmitt F (2003). p63: a novel myoepithelial cell marker in canine mammary tissues. *Veterinary Pathology*.

[8] De Los Monteros AE, Millán MY, Ramírez GA, Ordás J, Reymundo C, Martín De Las Mulas J (2005). Expression of maspin in mammary gland tumors of the dog. *Veterinary Pathology*.

[9] Ramalho LNZ, Ribeiro-Silva A, Cassali GD, Zucoloto S (2006). The expression of p63 and cytokeratin 5 in mixed tumors of the canine mammary gland provides new insights into the histogenesis of these neoplasms. *Veterinary Pathology*.

[10] Bertagnolli AC, Cassali GD, Genelhu MCLS, Costa FA, Oliveira JFC, Gonçalves PBD (2009). Immunohistochemical expression of p63 and *δ*Np63 in mixed tumors of canine mammary glands and its relation with p53 expression. *Veterinary Pathology*.

[11] Hampe JF, Misdorp W (1974). Tumours and dysplasias of the mammary gland. *Bulletin of the World Health Organization*.

[12] Moulton JE (1990). Tumors of the mammary gland. *Tumors in Domestic Animals*.

[13] Benjamin SA, Lee AC, Saunders WJ (1999). Classification and behavior of canine mammary epithelial neoplasms based on life-span observations in Beagles. *Veterinary Pathology*.

[14] Jabara AG (1960). Canine mixed tumours. *The Australian Veterinary Journal*.

[15] Sittner G (1939). Mammamischtumor bei einem männlichen Hund und seine Histogenese. *Archiv für Wissenschaftliche und Praktische Tierheilkunde*.

[16] Cotchin E (1947). Some glandular tumours of the dog. *Proceedings of the Royal Society of Medicine*.

[17] Nieberle K (1933). Zur Kenntnis der sog. Mammamischgeschwülste des Hundes. *Journal of Cancer Research And Clinical Oncology*.

[18] Mulligan RM (1949). *Neoplasms of the Dog*.

[19] Richards HG, McNeil PE, Thompson H, Reid SWJ (2001). An epidemiological analysis of a canine-biopsies database compiled by a diagnostic histopathology service. *Preventive Veterinary Medicine*.

[20] Genelhu MCLS, Cardoso SV, Gobbi H, Cassali GD (2007). A comparative study between mixed-type tumours from human salivary and canine mammary glands. *BMC Cancer*.

[21] Priester WA (1979). Occurrence of mammary neoplasms in bitches in relation to breed, age, tumour type, and geographical region from which reported. *Journal of Small Animal Practice*.

[22] Cavalcanti MF (2006). *Fatores prognósticos na abordagem clínica e histopatológica dos carcinomas mamários de cadelas: estadiamento TNM e sistema de Nottingham [M.S. thesis]*.

[23] Yamagami T, Kobayashi T, Takahashi K, Sugiyama M (1996). Prognosis for canine malignant mammary tumors based on TNM and histologic classification. *The Journal of Veterinary Medical Science*.

[24] Misdorp W, Cotchin E, Hampe JF, Jabara AG, von Sandersleben J (1972). Canine malignant mammary tumours. II. Adenocarcinomas, solid carcinomas and spindle cell carcinomas. *Veterinary Pathology*.

[25] Misdorp W (2002). Tumors of the mammary gland. *Tumours in Domestic Animals*.

[26] Bostock DE (1975). The prognosis following the surgical excision of canine mammary neoplasms. *European Journal of Cancer and Clinical Oncology*.

[27] Bertagnolli AC, Ferreira E, Dias EJ, Cassali GD (2011). Canine mammary mixed tumours: immunohistochemical expressions of EGFR and HER-2. *Australian Veterinary Journal*.

[28] Man YG, Tai L, Barner R (2003). Cell clusters overlying focally disrupted mammary myoepithelial cell layers and adjacent cells within the same duct display different immunohistochemical and genetic features: implications for tumor progression and invasion. *Breast Cancer Research*.

[29] Los de Monteros AE, Millán MY, Ordás J, Carrasco L, Reymundo C, Martín Las de Mulas J (2002). Immunolocalization of the smooth muscle-specific protein calponin in complex and mixed tumors of the mammary gland of the dog: assessment of the morphogenetic role of the myoepithelium. *Veterinary Pathology*.

[30] Man YG, Sang QXA (2004). The significance of focal myoepithelial cell layer disruptions in human breast tumor invasion: a paradigm shift from the "protease-centered" hypothesis. *Experimental Cell Research*.

[31] Gudjonsson T, Rønnov-Jessen L, Villadsen R, Rank F, Bissell MJ, Petersen OW (2002). Normal and tumor-derived myoepithelial cells differ in their ability to interact with luminal breast epithelial cells for polarity and basement membrane deposition. *Journal of Cell Science*.

[32] Xu Z, Wang W, Deng CX, Man YG (2009). Aberrant p63 and WT-1 expression in myoepithelial cells of pregnancy-associated breast cancer: implications for tumor aggressiveness and invasiveness. *International Journal of Biological Sciences*.

[33] Hinrichs U, Rutteman GR, Nederbragt H (1999). Stromal accumulation of chondroitin sulphate in mammary tumours of dogs. *British Journal of Cancer*.

[34] Erdélyi I, Nieskens DHM, Van Dijk JE, Vass L, Nederbragt H (2003). Immunohistochemical evaluation of versican, in relation to chondroitin sulphate, in canine mammary tumours. *Histology and Histopathology*.

[35] Erdélyi I, Van Asten AJAM, Van Dijk JE, Nederbragt H (2005). Expression of versican in relation to chondrogenesis-related extracellular matrix components in canine mammary tumors. *Histochemistry and Cell Biology*.

[36] Allen AC (1940). So-called mixed tumors of the mammary gland of dog and man. *Archives of Pathology*.

[37] Huggins C, Moulder PV (1944). Studies of the mammary tumours of dog. I. Lactation and the influence of ovariectomy and suprarenalectomy thereon. *The Journal of Experimental Medicine*.

[38] Bloom F (1954). *Pathology of the Dog and Cat: The Genitourinary System With Clinical Considerations*.

[39] Cotchin E (1958). Mammary neoplasms of the bitch. *The Journal of Comparative Pathology and Therapeutics*.

[40] Erichsen S (1955). A histochemical study of mixed tumors of the canine mammary gland. *Acta Pathology and Microbiology Scandinavica*.

[41] Hurley JV, Jabara AG (1964). Properties of “cartilage” in canine mammary tumors. *Archives of Pathology*.

[42] Pulley LT (1973). Ultrastructural and histochemical demonstration of myoepithelium in mixed tumors of the canine mammary gland. *American Journal of Veterinary Research*.

[43] Tateyama S, Cotchin E (1977). Alkaline phosphatase reaction of canine mammary mixed tumours: a light and electron microscopic study. *Research in Veterinary Science*.

[44] Tateyama S, Cotchin E (1978). Electron microscopic observations on canine mixed mammary tumors, with special reference to cytoplasmic filamentous components. *American Journal of Veterinary Research*.

[45] Destexhe E, Lespagnard L, Degeyter M, Heymann R, Coignoul F (1993). Immunohistochemical identification of myoepithelial, epithelial, and connective tissue cells in canine mammary tumors. *Veterinary Pathology*.

[46] Arai K, Nakano H, Shibutani M, Naoi M, Matsuda H (2003). Expression of class II *β*-tubulin by proliferative myoepithelial cells in canine mammary mixed tumors. *Veterinary Pathology*.

[47] Arai K, Uehara K, Nagai Y (1989). Expression of type II and type XI collagens in canine mammary mixed tumors and demonstration of collagen production by tumor cells in collagen gel culture. *Japanese Journal of Cancer Research*.

[48] Arai K, Uehara K, Nagai Y (1995). Simultaneous expression of type IX collagen and an inhibin-related antigen in proliferative myoepithelial cells with pleomorphic adenoma of canine mammary glands. *Japanese Journal of Cancer Research*.

[49] Tateyama S, Uchida K, Hidaka T, Hirao M, Yamaguchi R (2001). Expression of bone morphogenetic protein-6 (BMP-6) in myoepithelial cells in canine mammary gland tumors. *Veterinary Pathology*.

[50] Akiyoshi T, Uchida K, Tateyama S (2004). Expression of bone morphogenetic protein-6 and bone morphogenetic protein receptors in myoepithelial cells of canine mammary gland tumors. *Veterinary Pathology*.

[51] Kawabata A, Okano K, Uchida K, Yamaguchi R, Hayashi T, Tateyama S (2005). Co-localization of chondromodulin-I (ChM-I) and bone morphogenetic protein-6 (BMP-6) in myoepithelial cells of canine mammary tumors. *The Journal of Veterinary Medical Science*.

[52] Arai K, Naoi M, Uehara K (1994). Immunohistochemical examination of neural cell adhesion molecule (NCAM), tenascin and fibronectin on the development of cartilaginous tissue in canine mammary mixed tumors. *The Journal of Veterinary Medical Science*.

[53] Cassali GD, Bertagnolli AC, Gärtner F, Schmitt F (2011). Canine mammary tumours: a quantitative DNA study using static cytometry. *Revista Espanola de Patologia*.

[54] Bertagnolli AC, Soares P, van Asch B (2009). An assessment of the clonality of the components of canine mixed mammary tumours by mitochondrial DNA analysis. *The Veterinary Journal*.

[55] Ferletta M, Grawé J, Hellmén E (2011). Canine mammary tumors contain cancer stem-like cells and form spheroids with an embryonic stem cell signature. *The International Journal of Developmental Biology*.

[56] Voz ML, Van de Ven WJ, Kas K (2000). First insights into the molecular basis of pleomorphic adenomas of the salivary glands. *Advances in Dental Research*.

[57] Auclair PL, Ellis GL (1996). Atypical features in salivary gland mixed tumors: their relationship to malignant transformation. *Modern Pathology*.

[58] Ferreira E, Bertagnolli AC, Cavalcanti MF, Schmitt FC, Cassali GD (2009). The relationship between tumour size and expression of prognostic markers in benign and malignant canine mammary tumours. *Veterinary and Comparative Oncology*.

[59] Lewis JE, Olsen KD, Sebo TJ (2001). Carcinoma ex pleomorphic adenoma: pathologic analysis of 73 cases. *Human Pathology*.

[60] Wargotz ES, Norris HJ (1989). Metaplastic carcinomas of the breast. I. Matrix-producing carcinoma. *Human Pathology*.

[61] Livolsi VA, Perzin KH (1977). Malignant mixed tumors arising in salivary glands. I. Carcinoma arising in benign mixed tumors: a clinicopathologic study. *Cancer*.

[62] de Araújo VC, Altemani A, Furuse C, Martins MT, de Araújo NS (2006). Immunoprofile of reactive salivary myoepithelial cells in intraductal areas of carcinoma ex-pleomorphic adenoma. *Oral Oncology*.

[63] Chhieng C, Cranor M, Lesser ME, Rosen PP (1998). Metaplastic carcinoma of the breast with osteocartilaginous heterologous elements. *American Journal of Surgical Pathology*.

[64] Yamamoto Y, Kishimoto Y, Wistuba II (1998). DNA analysis at p53 locus in carcinomas arising from pleomorphic adenomas of salivary glands: comparison of molecular study and p53 immunostaining. *Pathology International*.

[65] Morris JS, Nixon C, King OJA, Morgan IM, Philbey AW (2009). Expression of TopBP1 in canine mammary neoplasia in relation to histological type, Ki67, ER*α* and p53. *The Veterinary Journal*.

[66] Deguchi H, Hamano H, Hayashi Y (1993). c-myc, ras p21 and p53 expression in pleomorphic adenoma and its malignant form of the human salivary glands. *Acta Pathologica Japonica*.

[67] Di Palma S, Skálová A, Vanìèek T, Simpson RHW, Stárek I, Leivo I (2005). Non-invasive (intracapsular) carcinoma ex pleomorphic adenoma: recognition of focal carcinoma by HER-2/neu and MIB1 immunohistochemistry. *Histopathology*.

[68] Matsubayashi S, Yoshihara T (2007). Carcinoma ex pleomorphic adenoma of the salivary gland: an immunohistochemical study. *European Archives of Oto-Rhino-Laryngology*.

[69] Gauchotte G, Coffinet L, Schmitt E (2011). Salivary gland anlage tumor: a clinicopathological study of two cases. *Fetal & Pediatric Pathology*.

[70] do Prado RF, Consolaro A, Taveira LA (2006). Expression of betacatenin in carcinoma in pleomorphic adenoma, pleomorphic adenoma and normal salivary gland: an immunohistochemical study. *Medicina Oral, Patología Oral y Cirugía Bucal.*.

